# Environmental factors influencing women’s childbirth experiences in labor–delivery–recovery–postpartum unit: a qualitative cross-sectional study

**DOI:** 10.1186/s12884-023-05488-7

**Published:** 2023-03-13

**Authors:** Ashraf Kazemi, Marjan Beigi, Hajar Enteshary Najafabadi

**Affiliations:** 1 Nursing and Midwifery Care Research Center, School of Nursing and Midwifery, Isfahan University of Medical Sciences, Isfahan, Iran; 2 Reproductive Health Department, School of Nursing and Midwifery, Isfahan University of Medical Sciences, Hezarjerib Av., Isfahan, Iran; 3 Student Research Committee, School of Nursing and Midwifery, Isfahan University of Medical Sciences, Isfahan, Iran

**Keywords:** Childbirth, Labor, Maternity, Qualitative research

## Abstract

**Background:**

Women’s experiences of birth environment influence their mental health and that of their families. Identifying women’s childbirth experiences in the labor–delivery–recovery–postpartum unit (LDRP) unit can help design a peaceful environment. Therefore, this study aimed to evaluate environmental factors influencing women’s childbirth experiences in LDRP unit.

**Methods:**

This qualitative cross-sectional study was conducted on 20 women with a childbirth experience in the LDRP unit. A purposive sampling was performed and continued until data saturation. The data were collected through unstructured interviews and analyzed using inductive content analysis.

**Results:**

Data analysis led to the extraction of three categories: physical security, a meaning-oriented environment, and physical comfort. The physical security category was obtained from three sub-categories: privacy, bed ergonomics, and the possibility of medical interventions. The meaning-oriented environment category was extracted from four sub-categories: promising symbols of becoming a mother, a peaceful environment, and a spiritual environment, and the physical comfort category was extracted from three sub-categories: minimizing noise pollution, ambient lighting, and LDRP internal design.

**Conclusions:**

These study results showed that women’s experience of giving birth in LDRP was accompanied by perceiving physical security, a meaning-oriented environment, and physical comfort. Moreover, the results indicated that the childbirth experience in the LDRP unit might be influenced by physical and emotional environmental factors. Therefore, in order to design a peaceful environment, it is necessary to take into account these factors.

## Background

Giving birth is a unique experience affecting women’s [1] and, accordingly, the family’s health [2]. A satisfactory vaginal birth is associated with a sense of self-efficacy for child care [3], successful breastfeeding [4], and more effective mother-child bonding [5]. However, the negative childbirth experience is correlated with the mother’s dissatisfaction with childbirth, the perception of trauma [6], unsuccessful breastfeeding [7], and increased probability of postpartum depression [8].

In addition, the mother’s increased stress and anxiety due to a negative experience during labor [9] can be associated with an increase in the need for sedatives during labor, slow progress of labor [10], and the baby’s low Apgar score [11]. Moreover, stress during childbirth is accompanied by an increased possibility of postpartum depression [8] and a disturbed orexinergic system, followed by disturbed mother-infant attachment [5] and delayed initiation of breastfeeding [7].

In addition to care safety, patient-centeredness is one of the main characteristics of quality care [12]. Therefore, maintaining mothers’ dignity and creating a satisfactory childbirth experience have been emphasized to promote their health and safe motherhood [13].

In a study, women described the childbirth experience as the senses, such as fear, anxiety, helplessness, and imminent death [14]. Women described the psychological trauma of childbirth as perceiving threats, prioritizing medical orders over the parturient women’s needs, and being ignored by the medical team [15]. Jordanian women described giving birth in a technologically processed environment as an inappropriate childbirth environment lacking human support [16]. These results show that although the development and application of technology have been associated with reducing pregnant women’s mortality rate [17], there remain obstacles that need to be identified to provide safe maternity services while maintaining human dignity and providing a positive childbirth experience.

A study has shown that the delivery environment was the main factor contributing to satisfaction with labor [18]. Martins et al. likewise reported that a non-abusive and respectful environment was one of the factors affecting women’s satisfaction with childbirth. Moreover, from the laboring women’s point of view, focusing on the labor environment and interpersonal relationships are of particular importance [19].

Another study similarly showed that women’s labor priorities included attention and physical care, emotional support, and the capability to transition into motherhood. However, healthcare providers prioritize breastfeeding and self-care education [20]. These findings show that women’s needs to experience a satisfactory delivery go beyond the provision of routine care by birth attendants and health care providers.

Although in an attempt to reduce the preventable risk factors of pregnancy and childbirth complications, access to medical technologies has been associated with reduced childbirth complications in complicated pregnancies [17], the extensive use of unnecessary equipment did not provide more satisfaction for maternity service recipients in uncomplicated pregnancies and deliveries [21].

In most countries, homelike environment/family-centered care units are designed for uncomplicated deliveries. The evidence shows that despite the global efforts toward the development of physiologic delivery in the maternity unit, the design of the delivery environment still needs reforms. A study in Nigeria showed that the staff’s clamor to control the situation transferred the staff’s stress to the parturient women [22].

In Iran, there are rooms called LDRP in the delivery department of hospitals in which the various stages of childbirth to discharge are carried out. A spouse or a companion is allowed in these units. The purpose of establishing these units was to create spaces similar to mother-friendly units in developed countries and to increase mothers’ satisfaction with natural childbirth.

Safe and satisfying delivery can be accurately planned by studying women’s natural childbirth experiences. Accordingly, the present study aimed to investigate environmental factors influencing women’s childbirth experiences in LDRP unit in a qualitative cross-sectional study.

## Method

This qualitative cross-sectional study, maintaining the psycho-social philosophy, was conducted in Isfahan from July to December 2021. The participants were 20 Iranian women who gave birth in the LDRP unit for the first time and were within 4–24 hours’ postpartum period following their vaginal delivery; as soon as the woman felt well and could communicate effortlessly.

Only participants with low-risk pregnancies and no severe mental illnesses based on their medical records were included in the study.

Purposive sampling was performed with maximum diversity in terms of age, education level, number of previous births, and duration of the active phase of the first stage of labor in three centers, including a university teaching hospital, a public non-teaching hospital, and a private hospital.

The selected hospitals had active LDRP units, and the treatment deputy of Isfahan had approved the feasibility of performing physiological childbirth in them. Each hospital included at least 2 LDRP units and was used for low-risk deliveries regardless of the number of previous pregnancies. The presence of a spouse or a companion was required. The equipment of each LDRP should at least include a delivery bed that could be converted into a regular bed, a baby cut, necessary delivery equipment, a fetal heart monitoring and resuscitation kit, a chair for the companion, and posters of the baby. Aromatherapy was also used in some hospitals. After selecting the hospitals, the inclusion criteria for LDRP patients were evaluated, and at least five eligible individuals were selected from each hospital.

Moreover, for each age group (below 25 years, 20 to 30 years, and over 30 years), education (diploma, bachelor’s, and higher), duration of active phase (less than 3 hours and more than 3 hours), and the number of previous births (zero, one, two and more than two), at least three individuals were included in the study.

After selecting the participant and explaining the study objective by one of the female researchers who was a reproductive health specialist, participation in the study was explained to them, and they were assured that in case of unwillingness to participate or continue the participation, their decision would not affect their received care. It was also explained that their information would be kept confidential, and the interview transcriptions would be accessible only to researchers. The participants were free to withdraw from the study at any stage. Informed verbal consent was obtained from all the invited individuals.

Data collection was initially conducted using semi-structured interviews using guiding questions. These guiding questions were developed based on three unstructured in-depth interviews. The followings are examples of guiding questions: “Explain your feelings upon entering this environment” and “Explain memorable things during childbirth.” During the interview, an attempt was made to obtain a better understanding of the experiences by using questions such as “Please explain more about your feelings” and recording emotional reactions and non-verbal expressions.

The interviewer was one of the female researchers, who was a postgraduate midwifery student, had participated in the “Data Collection in Qualitative Studies” workshop, and had conducted two probationary interviews supervised by a person experienced in conducting qualitative research in the field of reproductive health.

The interviews were conducted in the LDRP unit when care providers were not present. The interviews were tape-recorded; however, they were transcribed in case of the participant’s reluctance to be voice-recorded. Permission was obtained before initiating the recording.

Considering each participant’s conditions, the interview lasted approximately 60 to 90 minutes. After each interview, the researcher listened to the interviews several times to gain a comprehensive understanding of the interviews. Afterward, the interviews were transcribed verbatim. The participants’ emotional reactions and recorded non-verbal expressions were recorded next to the related text. Data analysis was performed simultaneously with each interview. The interviews continued until data saturation was reached when no new inferential codes and sub-categories emerged.

Qualitative content analysis was performed manually by the interviewer through an inductive approach and using the Graneheim and Lundman method [23]. Qualitative content analysis is a suitable method for data analysis in studies aiming to describe important subjects for specific individuals [24]. Since individuals’ experiences are interpreted in the context in which they live, this analysis method was selected. Moreover, since available information about the subject under investigation was insufficient, the inductive approach was suitable for data analysis [25].

The stages of data analysis included determining the content, unit of analysis and meaning units, concision and summarization, code formation and categorization, and formation of sub-categories and main categories. Each interview was considered a unit of analysis.

After transcribing the interview, it was read several times, and meaning units and primary codes were extracted. In the following step, inferential codes were formed, and similar codes were merged. Similar inferential codes were placed in the sub-categories, and the sub-categories and the main categories were inferred.

To ensure the trustworthiness of the data, four criteria, including credibility, dependability, transferability, and conformability, were evaluated. To increase the validity of the findings, the method of checking the findings and the external check were used. In this way, the congruity of the findings with experiences was assessed by calling 3 participants.

Moreover, the results and analysis of the findings were provided to three midwifery experts, who were not the research team members, and their complementary and critical comments were considered. For dependability, all research stages were recorded and presented as a report on the results of some interview transcriptions. The purposive sampling method with maximum diversity was used to meet transferability. In order to increase conformability, all the stages of the study were recorded, and documents and reports were maintained.

## Results

In this study, 21 women who gave birth in the LDRP unit were invited to an interview. Totally, 20 individuals aged 21–35 years with 1–6 pregnancies participated in the study, and one individual refused to participate. The participants’ education ranged from diploma to Ph.D.; their characteristics are presented in Table 1. The analysis of 20 interviews led to the extraction of 828 inferential codes, 25 sub-sub-categories, nine sub-categories, and three main categories. The main categories included physical security, a meaning-oriented environment, and physical comfort (Table 2).

**Table 1:** Demographic characteristics of the participants

Participant’s Number	Age	Educational level	Employment status	Pregnancy number
P1	24	Diploma	Housewife	One
P2	23	Diploma	Employed	One
P3	34	Diploma	Employed	Three
P4	32	Diploma	Housewife	One
P5	29	Diploma	Housewife	Two
P6	28	Bachelor’s degree	Housewife	Six
P7	28	Diploma	Housewife	One
P8	27	Bachelor’s degree	Housewife	One
P9	20	Diploma	Housewife	One
P10	23	Diploma	Employed	One
P11	29	Diploma	Employed	Two
P12	26	Diploma	Housewife	Two
P13	31	PhD	Employed	Two
P14	35	Bachelor’s degree	Housewife	Two
P15	26	Bachelor’s degree	Housewife	One
P16	22	Master’s degree	Employed	Three
P17	30	Master’s degree	Employed	One
P18	25	PhD	Employed	Two
P19	32	Bachelor’s degree	Housewife	One
P20	26	Master’s degree	Housewife	One

**Table 2:** The main categories, and sub categories

Main Category	Subcategory
Physical Security	Privacy
	Bed Ergonomics
	Possibility of Medical Interventions
Meaning-oriented Environment	Promising Symbols of Becoming a Mother
	Peaceful Environment
	Spiritual Environment
Physical Comfort.	Minimizing Noise Pollution
	Ambient Lighting
	LDRP Internal design

*Abbreviations: LDRP* labor–delivery–recovery–postpartum

### Physical security

This main category was derived from the sub-categories of privacy, bed ergonomics, and the possibility of medical interventions.

#### Privacy

The privacy sub-category was formed based on the statements indicating participants’ need for privacy in the LDRP environment. The data showed that one of the participants’ concerns during labor was that others might observe them during the examination or delivery, and they preferred that the physical conditions of the LDRP unit minimized the possibility of the lower body, especially the genital area, being observed. Participant No. 2 said: “*My husband was by my side during childbirth. He was sitting where he could see my genital area. It upset me. I didn’t like him to see my body during childbirth. I wanted his face to be near my face*.”

Participant No. 9 stated: “*After giving birth, I wished there had been a private environment where I could talk to my baby; look at her; sing a song to her, but I was shy. I was worried someone would pass by the room and hear me chanting. The door was open, and the staff asked me not to close it*.

#### Bed ergonomics

Another sub-category related to physical security was bed ergonomics. This sub-category was extracted from statements pointing to the characteristics of the delivery bed and focused specifically on the sense of stability on the bed, ease of changing position, protective railings, and the possibility of viewing the baby in the cot. Some statements indicated that the conditions in which a laboring mother might slip off the bed during the second stage as she made an effort to push the baby out caused a sense of instability and falling.

In this regard, Participant No. 3 said: “*It was as if the bed was sloping downwards. I was worried that I would fall off the bed when pushing the baby out or that the baby would be born and fall. I wanted to pull myself up, but I could barely do it*.”

The statements of a significant number of participants (10 individuals) pointed to the importance of visiting the baby immediately after delivery; however, the bed position hindered their view. The participants expected the bed position to provide them with an easy view of the baby after delivery to ensure his/her health. In this regard, participant No. 5 said: “*It was very good that the baby’s bed was next to mine, but when the baby was born, and the midwives were cutting the umbilical cord, I couldn’t see my baby. I kept raising my head, but actually, the placenta wasn’t born yet, and I had to lie down again. It would be great if the bed was in such a way that I could see the baby in the same position*.”

#### The possibility of medical interventions

The possibility of medical interventions was another sub-category of the physical security category, derived from the ‘medical equipment in the unit’ and ‘suitable environment for medical interventions’ sub-categories. The possibility of medical interventions was formed especially based on the statements of the participants who had previously experienced a complicated pregnancy and childbirth.

The participants expected medical interventions to be available in case of problems. Regarding that, participant No. 6 said: “*When I entered the room, I noticed that the room was well-equipped. I felt relieved. I was assured they wouldn’t need to take it from other places or move me if something went wrong. They would help me here*.” Participant No. 20 said: *The room size is important. Having all equipment available is great, but I wish they weren’t so visible to make the room cramped. You feel short of breath in this small space with many things*.”

### The meaning-oriented environment

This category was formed from the sub-categories of promising symbols of becoming a mother, a peaceful environment, and a spiritual environment.

#### Promising symbols of becoming a mother

This sub-category was derived from statements indicating that the baby’s bed was a promise to become a mother, and the fetus’s images and heart sound created the sense of becoming a mother.

The results showed that some elements in the LDRP unit promised the imminent birth of a baby and motherhood. The baby’s cot next to the mother’s bed before birth caused exhilaration to mothers. For some participants, the baby’s cot next to the mother’s bed made them visualize their baby before giving birth.

Regarding this, participant No. 1 said: “*As soon as I entered the room, I noticed the baby’s bed. I unconsciously imagined my baby in bed. It feels good. I believed that my waiting was ending and that the baby was coming. It was perfec*t.”

Baby photo LDRP wallpapers increased the birth excitement for some participants. In addition, playing the sound of the fetus’s heartbeats added to these feelings. In this regard, participant No. 9 said: “*When I heard the sound of the baby’s heartbeat, I felt it with my whole being. It was a motivating feeling. I had heard this sound a lot before. But the sound of his heart was different when he was coming. It was as if he was telling me that he was coming. I felt that my child was healthy. He would be born soon, and I become a mother, mother of a healthy and roly-poly child* (smiling).”

#### A peaceful atmosphere

This sub-category was the result of analyzing participants’ positive and negative experiences of hearing the music and smelling the scent in the LDRP environment. Playing the music that coordinated the participant’s preference provided a pleasant feeling. On the contrary, playing music that was not their preferred type lowered their pain threshold. Regarding this, participant No.5 said: “*I didn’t like the music playing. It was on my nerves. I liked the softer music. I wish the poems of Rumi (an Iranian poet) would be played so they would be worth listening to. Playing music is not the only important point. It should be something that you like to listen to and can focus on it. (While giving birth) I wished I had brought headphones to listen to my favorite music*.”

Using pleasant scents was one of the participants’ demands inferred from their interviews.

Participant No. 21 said: “*The room smelled disinfectants. It was better If it smelled a scent, so you would feel that you were in a natural environment like your home and felt healthier. But in this situation (the smell of antiseptics), I assumed I was sick and visited the hospital*.”

#### A spiritual environment

Two comforting sounds for some participants with religious beliefs (Islam) included the sound of the Qur’an (the holy book of Muslims) recitations before giving birth and the Muslim call to prayer (a voice in which God is praised and whispered in the baby’s ear after birth) after the baby’s birth. Regarding this, some participants stated that hearing the sound of the Qur’an helped them tolerate pain. Participant No.8 said: “*I had brought a music player with me; I played the Qur’an recitation, and when I heard it, I felt that someone (God) was with me. He was kind and would help me. I was in pain, but I didn’t think about i*t.”

Participant No.3 stated: “*Immediately after giving birth, the call to prayer was played. When he said Allahu Akbar (a part of the Muslim adhan that means God is the greatest), I was reciting along. That was how I thanked God for my child and me being safe and sound*.”

### Physical comfort

This category was derived from the sub-categories minimizing noise pollution, ambient lighting, and environment design and color.

#### Minimizing noise pollution

This sub-category was inferred from statements about electromagnetic interference, continuous playback of fetal heartbeat sounds, and sounds outside the LDRP unit. The analysis of the experiences of the participating women showed that some women were annoyed by environmental sounds, including noise from electronic devices, such as Sonicaid and fetal heart monitoring. Moreover, the sudden changes in the fetal heart rhythm and the decrease in the sound of the devices when playing the sound of the fetal heartbeat, as well as the electromagnetic interference, caused discomfort to the mother. Participant No. 3 said: “*Hearing the sound of the baby’s normal heartbeat comforted me, but when the baby was moving, and the sound (of the monitor) was distorted, I was afraid. I worried about why it made such a sound. Sometimes it was choppy. The midwife told me not to worry because the device wasn’t stable, but I was*.”

In addition, the noise of the staff in emergencies to help other patients and the sound of the mothers in labor pain caused discomfort to participants. Regarding this, participant No. 6 said: “*Midwives’ talking and laughing sounds outside the room relaxed me. I thanked God that no one had any problem. But when I heard other patients screaming, I was scared and stressed. I hoped she would give birth soon*.”

Participant No. 15 said: “*When I heard other laboring women’s yells, I forgot my own pain. I felt sorry for them. Their pain was like my pain. I could reduce my pain with the exercises I had already learned. But I could do nothing for others*.”

#### Ambient lighting

This sub-category was derived from concepts that indicated the participants’ preference for sufficient lighting in the LDRP unit. Most participants considered the direct ceiling lighting irritating. Regarding this, participant No. 9 said: “*I was in pain, and the ceiling light was directly hitting my eyes. I couldn’t sleep in the supine position*.”

Participant No.2 said: “*I felt suffocated when I entered the room. The room was poorly lit. If it were lighter, I would feel safer. I entered a new environment that was also dark. Honestly, I was a little scared, but later I got used to it*.”

#### Internal design of LDRP

This sub-category was formed from statements implying that participants preferred bright, energizing colors and design appropriate for mother and child rooms. This sub-category was inferred from the sub-sub-categories, indicating the importance of the walls’ and curtains’ color and the orderly arrangement of the room furniture. The brightly colored curtains with baby images were acceptable for the participants, and they preferred bright and neutral colors for walls.

Participant No. 12 said: “*It would be better to color the room wall white than green. The green color makes the room look small. You feel sleepy. The color of the curtains (pink and blue) was nice.*”

Participant No. 9 stated: “*The baby images on the curtains were an interesting idea. Looking at the baby’s face comforted me. I wished my child looked like this (laughter). Ohhh...; when I looked at these photos, I wanted to see my child. I was excited to see the baby*.”

The arrangement of LDRP devices was a sub-sub-category that formed the LDRP interior design sub-category and was inferred from experiences indicating the importance of the device arrangement for the participants. They believed the room condition needed to provide them with space for extra items, such as the companion’s items or medical equipment in special closets. Unarranged equipment in some rooms caused discomfort for the participants. In this regard, participant No. 7 said: “*when I entered the room, I felt a mess due to many items in the room. Some items, such as trash cans and oxygen equipment, could be out of sight. Well, they should be in the room, but better to be less visible. I’m very obsessive. I think everything should be neat and organized.*”

## Discussion

This qualitative study was conducted to more deeply understand the environmental factors influencing women’s childbirth experiences in LDRP unit in Iran. The results showed that women’s childbirth experiences in the LDRP could be discussed under three main categories: physical security, a meaning-oriented environment, and physical comfort. The physical security category was derived from the sub-categories: privacy, bed ergonomics, and the possibility of medical interventions.

The participants in this study considered privacy in the LDRP environment essential for their peace of mind. They stated that the protected environment was an issue that ensured their privacy. A study in Laos showed that the lack of privacy, the impossibility of following traditional practices in the hospital, and the presence of male staff were some barriers to giving birth in health centers [26].

The provision of respectful maternity services is a fundamental human right [13]. The results of a systematic review have shown that the provision of respectful maternity care requires privacy [27]. The results of the present study likewise confirm the need to respect privacy in providing respectful maternity services.

The ergonomics of the delivery bed was another sub-category from which the physical security category was derived. These results showed that women preferred the delivery bed to allow their preferred position. This finding confirms the results of the study by Sychareun et al., who reported that the compulsion to give birth horizontally was one of the obstacles to giving birth in health centers [26]. In addition, the results of the study showed that the labor bed was required to alleviate the fear of falls in different positions. Therefore, in designing the LDRP bed, the physical protection of women in different positions must be taken into account.

Another sub-category of physical security was the possibility of medical interventions. Although childbirth is a physiological process, its dynamic nature and the potential risky obstetric emergencies necessitate access to medical facilities. Ensuring access to essential interventions in the LDRP unit is undisputed for those who have experienced pregnancy and childbirth complications. It has been reported that concerns about childbirth complications compel some women to opt for hospitals equipped with medical intervention and facilities [28].

Furthermore, compared to other women, those with a history of birth injuries have reported the need for supportive care and access to medical equipment. Stevens et al. likewise reported that those women tended to give birth in hospitals equipped with medical technology [28].

However, an assessment of psychological conditions for childbirth in a study showed that women with low-risk pregnancies prefer to give birth in a place with less medical equipment [29]. The results of these studies [28, 29] and the present study show that access to medical equipment in LDRP is necessary, and women need to be assured of the necessary facilities for safe delivery. Ensuring access to essential interventions in the LDRP is more critical for those who have experienced complications of pregnancy and childbirth. Meanwhile, based on the present study, it can be recommended that medical equipment should be easily accessible for all delivery types in LDRP; however, it should not transform the LDRP environment into a technological environment.

The second category extracted was “a meaning-oriented environment,” inferred from the sub-categories: promising symbols of becoming a mother, a peaceful environment, and a meaning-oriented environment. These results showed that the elements in LDRP indicating the imminence of birth and the beginning of motherhood comforted the women in labor.

Locating the baby’s cot next to the mother’s bed and the fetus’s images were promising for the laboring woman to become a mother. Besides, the sound of the fetus’s heartbeats through a playback device strengthened their maternal emotions. In this regard, an ethnographic study in Germany showed that the feeling of fetal movement strengthened the perception of the fetus as a real being [30]. These results show that realistic symbols of the baby’s existence evoke maternal emotions and probably assist the process of maternal development.

One of the sub-categories of the meaning-oriented environment category was “a peaceful environment.” From the participants’ perspective, hearing music and smelling blossoms could create a relaxing atmosphere in LDRP. However, the remarkable point was that they preferred their favorite music to be played. Moreover, the scent of flowers created a pleasant experience if it were the participants’ preference. This finding is in line with the studies indicating that playing music in the delivery environment reduced the mother’s anxiety [31]. An interventional study has likewise shown that creating a homelike environment, such as watching nature images and TV during labor pains, was associated with improved fetal heart rate and Apgar score, reduced need for painkillers during labor, and the mother’s positive view of childbirth [32].

In another interventional study, listening to music has been shown to reduce labor pain and women’s anxiety during labor [33]. These finding suggests that the laboring woman’s preferences should be considered in providing services such as playing music and using the scent.

Another finding of the study, which led to the emergence of the meaning-oriented sub-category, was the Muslim call to prayer (Adhan) after the birth of a baby, which created a spiritual and comforting feeling in women. Reciting a part of Adhan that includes praising God in the baby’s ear is considered a religious ritual for Muslims. The effect of the sound of the Quran (Muslim holy book) on reducing Muslim women’s pain of delivery has been previously reported. A study on Muslim patients suffering from anxiety in Malaysia showed that using Holy Quran recitation through a cognitive behavioral therapy program was associated with reducing patients’ anxiety [34]. This effect has also been confirmed in a systematic review [35]. This finding of the present study shows that the integration of the birth process into spiritual tendencies can create a more peaceful environment in the LDRP unit.

The physical comfort category was another finding derived from the ‘noise pollution minimization, ambient lighting, and LDRP interior design’ sub-categories. The results show that for a peaceful LDRP, it is necessary to minimize noise pollution. In this study, noise pollution included other women yelling and screaming due to labor pain, the personnel’s tumult encountering an emergency and electromagnetic interference, particularly from the devices monitoring the fetal heartbeat, creating a sense of possible risk for the fetus.

Furthermore, the results showed that sufficient indirect lighting gave the laboring women a sense of security, whereas insufficient ambient lighting made them feel at risk. There are apparent reasons that sufficient lighting increases alertness and can induce a sense of control over the situation [36, 37]. These results of the present study indicated that the integration of the birth process into spiritual tendencies could create a more peaceful environment in the LDRP unit.

These results confirm the necessity of sufficient lighting in optimal conditions and quality for a peaceful LDRP.

Another sub-category that emerged from the data analysis was LDRP interior design, emphasizing revitalizing and bright colors, a design coordinating mother and child room, proper equipment arrangement, and mother’s access to baby.

The positive effect of early skin-to-skin contact between mother and baby on the mother’s anxiety and pain is documented [38] and recommended in clinical guidelines [39]. This study likewise indicated that mothers were interested in seeing their babies soon after birth, and it is necessary to provide them with the opportunity to see and access the baby at different stages of baby care.

This is the first study on the childbirth experience in LDRP in Iran, and its results can be used for designing LDRP according to the Iranian women’s culture and tendencies; however, for the interpretation and application of the results, the limitations of this study should be taken into account. The heterogeneity of the participants regarding childbirth experience is one of the limitations of this study since women’s experiences in LDRP can be affected by the experience of their previous births. In this study, although all the participants experienced giving birth in LDRP for the first time, they were dissimilar in terms of labor experience. These limitations included representativeness and sampling technique. Moreover, the short time after delivery (4 to 24 hours) was one of the limitations of the present study that could affect the results. Furthermore, the study was conducted during the COVID-19 pandemic, and concerns about disease transmission might have affected the results.

## Conclusion

These results showed that women’s experience was accompanied by perceiving physical security, a meaning-oriented environment, and physical comfort. The results indicated that the LDRP environment is required to provide comfort, peace, and security to meet the laboring women’s needs. For these needs to be met, laboring women’s privacy needs to be maintained, the LDRP environment should be designed to promise the baby’s birth, and noise pollution due to electromagnetic interference should be minimized as much as possible.

It is suggested that the results of this study be used in compiling the charter of mothers’ rights in the LDRP unit and the LDRP design, and their impact on childbirth satisfaction and the maintenance of women’s mental health be evaluated.

## Data Availability

Data and material are available on request from the corresponding author.

## Abbreviation

LDRP: labor delivery recovery postpartum
